# Treatment of cancer with cryochemotherapy

**DOI:** 10.1038/sj.bjc.6600306

**Published:** 2002-05-03

**Authors:** L M Mir, B Rubinsky

**Affiliations:** FRE 2530 CNRS, Institut Gustave Roussy, PR II, 39, rue Camille Desmoulins, F-94805 Villejuif Cédex, France; Department of Biomedical Engineering, 6105 Etcheverry Hall, University of California at Berkeley, Berkeley, California, CA 94720, USA; Department of Mechanical Engineering, 6105 Etcheverry Hall, University of California at Berkeley, Berkeley, California, CA 94720, USA

**Keywords:** cryosurgery, frozen tissue, bleomycin

## Abstract

Cryosurgery employs freezing to destroy solid tumours. However, frozen cells can survive and cause cancer recurrence. Bleomycin, an anticancer drug with a huge intrinsic cytotoxicity is normally not very effective because it is nonpermeant. We report that freezing facilitates bleomycin penetration into cells making it toxic to cryosurgery surviving cells at concentrations that are non-toxic systemically.

*British Journal of Cancer* (2002) **86**, 1658–1660. DOI: 10.1038/sj/bjc/6600306 www.bjcancer.com

© 2002 Cancer Research UK

## 

Cryosurgery is a minimally invasive surgical technique that employs freezing to destroy solid tumours. Deep in the body it is performed with needle-like cryogen cooled probes, inserted in the tumour. The extent of freezing is controlled by imaging (Onik *et al*, 1984, 1991; Rubinsky, 2000). The frozen tissue is left to thaw *in situ* and is disposed by the body immune system. During cryosurgery, freezing propagates from the cryosurgical probe outward. Cells in the frozen lesion experience a range of temperatures from cryogenic near the probe interface (lower than −140°C), to phase transformation on the outer edge (−0.56°C) (Rubinsky, 2000). On the outer rim of the frozen lesion, in the temperature range between −0.56°C and about −40°C, cells can survive (Rui *et al*, 1999; Rubinsky, 2000). Therefore, despite imaging, there is uncertainty in the application of the procedure and at times cryosurgery failure (Revoire *et al*, 1996).

Bleomycin is an anticancer drug with a huge intrinsic cytotoxicity. It is not very effective because it cannot enter cells freely (Mir *et al*, 1996; Pron *et al*, 1999). However, increasing cell membrane permeability with electrical procedures results in a huge increase in bleomycin cytotoxicity, (Belehradek *et al*, 1993; Mir *et al*, 1998; Kotnik *et al*, 2000; Mir, 2001). Our hypothesis was that the change in membrane permeability and increase in extracellular solute concentration during freezing (Mazur, 1970; Rubinsky, 2000) might facilitate penetration of bleomycin in frozen cells which could make it useful in treating cells on the outer rim of the frozen lesion. We tested the hypothesis by freezing cells with thermal parameters typical to the outer rim of the frozen lesion, in which cells survive freezing, in the presence of various doses of bleomycin (Rui *et al*, 1999; Rubinsky, 2000). This found that freezing facilitates bleomycin penetration into cells, making it toxic to cryosurgery surviving cells at concentrations that are normally non-toxic. Since both cryosurgery and bleomycin are routinely used for clinical treatment of cancer the combination should have immediate clinical utility.

## MATERIALS AND METHODS

Experiments were performed with B16 F0 melanoma cells (ATCC CRL 6322). First, 100 μl of S-MEM (Life Technologies, Rockville, MD, USA) in a 15 ml tube (Falcon, Becton Dickinson, Franklin Lakes, NJ, USA) were frozen in a −20°C freezer, in air, to produce an ice seed. Then 400 μl of cells just suspended in ice-cooled S-MEM with either 5 μM, 500 nm, 100 nm or 10 nm of bleomycin or without bleomycin were put in the test tubes with the frozen solution and inserted in the −20°C freezer. A T type thermocouple (Omega, Stamford, CT, USA) which was put in a thermal control sample and used with each experiment has shown that the solutions froze with a cooling rate of 1±0.2°C/min to a temperature of −14±1°C. The cells were exposed to freezing conditions for 30±4 min. The frozen samples were thawed by immersion in water at room temperature. They thawed within 10 min. Following thawing the samples were diluted with 2 ml of normal MEM culture medium (Life Technologies) supplemented with 8% foetal calf serum (Life Technologies) and penicillin and streptomycin (Sarbach/Solvay Pharma, Brussels, Belgium), which resulted in a five times dilution of the bleomycin concentration. Cells were transferred to 35 mm Petri dishes (TPP, Trasadingen, Switzerland) and incubated at 37°C and 5% CO_2_ (Universal Water Jacketed Incubator, Forma Scientific, Marietta, OH, USA). Overall viability of the cells was inspected visually immediately after and 3 to 4 h after the treatment. Six hours after the treatment (i.e. after cell attachment), medium was changed in the experiments in which cells were exposed to 5 μM bleomycin in order to reduce the external concentration of the drug to non-cytotoxic levels. In the case of the experiments with 500 nM, 100 nM or 10 nM bleomycin, the medium was only changed 24 h after the treatment (it has been shown that for all these concentrations a five times dilution is below the toxic concentration when cell exposure time to bleomycin or bleomycin analogues was 48 h or longer (Takahashi *et al*, 1987)). In half of the controls the medium was also changed after 6 h and in the other half after 24 h, to remove the cells killed by the freezing procedure. No statistical difference in the number of colonies was observed between these two groups of controls. After 137 h of culture the medium was removed, cells were fixed using 1.5 ml of formol 2% in NaCl 0.9% for 10 min, and then stained for 15 min using 2 ml of crystal violet (Labo Moderne, Paris, France) diluted to 1 : 5 in water. After extensive rinsing, colonies were manually counted under a binocular microscope Stemi SV6 (Zeiss, Le Pecq, France). Photographs were taken using a Contax 167MT camera (Zeiss) directly installed on an Axiovert 135 (Zeiss) inverted microscope, on 160 T Ektachrome Kodak films. Slides were scanned using a Mirage S2 scanner (UMAX). Statistical analysis was performed using ANOVA (One Way Analysis of Variance).

## RESULTS AND DISCUSSION

The apparent cell survival immediately after thawing, evaluated from cell membrane integrity and morphology, was low, typical to the tested freezing conditions and relatively similar in all the samples with no discernable trend. This is to be expected as the effect of bleomycin should be long term, to prevent cell division, and not immediately evident. Table 1

**Table 1:**
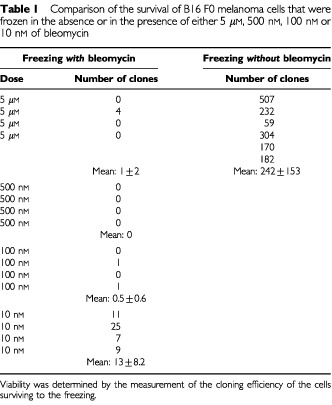
Comparison of the survival of B16 F0 melanoma cells that were frozen in the absence or in the presence of either 5 μM, 500 nM, 100 nM or 10 nM of bleomycin

compares the long-term survival of B16 F0 melanoma cells, in culture. Six experiments with cells frozen in S-MEM alone show that they produced a mean of 242±153 colonies. In contrast, bleomycin at concentrations of 5 μM, 500 nm and 100 nm caused, in four separate experiments for each concentration, a significant decrease in viability producing a mean of 1±2, 0, and 0.5±0.6 colonies, respectively (statistically different of the controls, *P*<0.001 using ANOVA test). The effect of bleomycin seems to taper off at a concentration of 10 nm, for which four experiments produced a mean of 13±8.2 colonies (statistically different of the three other bleomycin groups, *P*=0.002 using ANOVA test).

It is well established in the cryobiology literature that cells can survive freezing. There are various mechanisms of freezing damage and they are complex. On the outer edge of the frozen cryosurgical lesion, where cells survive, the mechanism of damage is due to ‘low’ cooling rates (Rubinsky and Shitzer, 1976; Rubinsky, 2000). Mazur (1970) explained that at what are considered ‘low’ cooling rates, ice forms first extracellularly, while the intracellular solution does not freeze and remains supercooled. Ice has a tight crystallographic structure and rejects the solutes in the unfrozen region, usually around the unfrozen cells. To equilibrate the difference in chemical potential across the cell membrane water leaves the cell to freeze in the extracellular space. The consequent cell dehydration causes an increase in intracellular cell osmolality. Cell dehydration together with cell membrane lipid phase transition and transmembrane ionic leakage combine to produce chemical damage to intracellular components. The range of temperatures in which the chemical damage takes place is between the phase transition temperature, −0.56°C, and the saline eutectic, about −32°C. While the mechanism of damage is the same in the entire temperature range, the extent of the damage increases with lower temperatures and with time of exposure. The thermal parameters employed in this study were chosen because they are typical of non-lethal freezing thermal parameters most relevant to the problems of cryosurgery. The cooling rate of 1°C/min is typical to ‘low’ cooling rates (Rubinsky and Shitzer, 1976, 2000), the temperature of −14°C is a typical temperature in which cell survival was observed and in which the mechanism of damage is chemical (Revoire *et al*, 1996; Rui *et al*, 1999; Rubinsky, 2000) and the time of exposure of 30 min is typical to how long a cryosurgical procedure takes (Onik *et al*, 1984, 1991).

**Figure 1:**
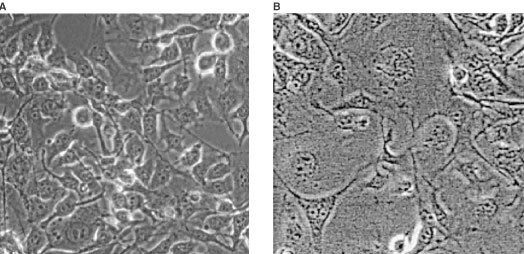
Typical morphological differences between B16 F0 melanoma cells that were frozen in the absence (**A**) or in the presence of 100 nm of bleomycin (**B**), after 137 h of incubation. (Magnification 1100×). In the absence of bleomycin, the surviving cells display a normal morphology, regular cell size, and the usual nucleus aspect. Normal mitosis can be observed in the two rounded cells on the right upper part of panel A. In contrast, in panel B cells show abnormal morphology. The size of the nuclei and cytoplasm has increased. The limits of the nucleus are not always demarcated while the cytoplasm itself is clear. Among cells there are large differences in nuclei and cell size.

Table 1 demonstrates that bleomycin has the ability to significantly increase cell damage at thermal conditions typical to those on the outer rim of a cryosurgical lesion. Bleomycin molecules form complexes with Fe^2+^ and O_2_ that directly generate breaks on DNA through the nucleophilic attack of the C′_4_ of the desoxyribose, particularly at the GC sequences (Povirk *et al*, 1989; Mir *et al*, 1996). Since bleomycin must penetrate the cell to affect cell survival, the data presented in Table 1 suggests that the bleomycin has entered the cells during freezing and that it is affecting cells which otherwise would have survived the freezing protocol. Figure 1

confirms this. It shows the appearance of cells grown after freezing with and without bleomycin, after 137 h of culture. The cells that survived the freezing procedure and were not exposed to bleomycin attached to the substrate and kept normal morphology as well as growth features (Figure 1A). On the contrary, in the presence of bleomycin at the time of cell freezing, the attached cells did not show mitoses and their morphology was very altered (Figure 1B). Moreover, these figures show ‘mitotic cell death’ typical of bleomycin internalisation (Tounekti *et al*, 1993, 2001).

While an anticancer drug with a huge intrinsic cytotoxicity (several hundred molecules of bleomycin inside the cell are sufficient to kill (Poddevin *et al*, 1991), mediated by the generation of DNA double-strand breaks), bleomycin is normally not very effective for treatment of cancer because it is nonpermeant (it enters cells not by diffusion through the plasma membrane (Poddevin *et al*, 1991) but rather by a mechanism of receptor-mediated endocytosis) (Mir *et al*, 1996; Pron *et al*, 1999). There is abundant evidence to show that concentrations of 500 nm and 100 nm bleomycin have no effect on cells (Barranco and Humphrey, 1971; Takahashi *et al*, 1987; Mir *et al*, 1996). This study demonstrates that bleomycin at doses as low as 100 nm, and even 10 nm, has the ability to significantly reduce the survival of cells frozen with thermal conditions typical of the outer rim of the cryosurgical frozen lesion. For the bleomycin to function at these low concentrations there must be a mechanism by which it can enter the cells during freezing. It is established that the cell membrane is permeabilised during cooling and freezing due to such mechanisms as lipid phase transition, dehydration induced membrane deformation and ice crystal induced membrane deformation (Mazur, 1970; Quinn, 1985). In addition, as discussed earlier, because ice cannot contain solutes, the effect of freezing is to reject all the solutes in the unfrozen region, around the cells. This usually increases the ionic concentration around the cell; however, when bleomycin is present in the solution the concentration of bleomycin near the cell will also increase. Therefore we propose that the temporary increase in membrane permeability during cooling and freezing, as well as the freezing-induced increase in bleomycin concentration around cells will cause the bleomycin to enter cells during cryosurgery, when used at doses that otherwise would have no effect on the cells.
